# A Novel Signature Constructed by Immune-Related LncRNA Predicts the Immune Landscape of Colorectal Cancer

**DOI:** 10.3389/fgene.2021.695130

**Published:** 2021-08-09

**Authors:** Mengyu Sun, Tongyue Zhang, Yijun Wang, Wenjie Huang, Limin Xia

**Affiliations:** ^1^Department of Gastroenterology, Institute of Liver and Gastrointestinal Diseases, Tongji Hospital of Tongji Medical College, Huazhong University of Science and Technology, Wuhan, China; ^2^Hepatic Surgery Center, Tongji Hospital, Tongji Medical College, Huazhong University of Science and Technology, Wuhan, China; ^3^Hubei Key Laboratory of Hepato-Pancreato- Biliary Diseases, Tongji Hospital, Tongji Medical College, Huazhong University of Science and Technology, Wuhan, China

**Keywords:** colorectal cancer, immune-related lncRNA, immunotherapy, signature, TCGA

## Abstract

Colorectal cancer (CRC) has the characteristics of high morbidity and mortality. LncRNA not only participates in the progression of CRC through genes and transcription levels, but also regulates the tumor microenvironment and leads to the malignant phenotype of tumors. Therefore, we identified immune-related LncRNAs for the construction of clinical prognostic model. We searched The Cancer Genome Atlas (TCGA) database for original data. Then we identified differentially expressed irlncRNA (DEirlncRNA), which was paired and verified subsequently. Next, univariate analysis, Lasso and Cox regression analysis were performed on the DEirlncRNA pair. The ROC curve of the signature was drawn, and the optimal cut-off value was found. Then the cohort was divided into a high-risk and a low-risk group. Finally, we re-evaluated the signature from different perspectives. A total of 16 pairs of DEirlncRNA were included in the construction of the model. After regrouping according to the cut-off value of 1.275, the high-risk group showed adverse survival outcomes, progressive clinicopathological features, specific immune cell infiltration status, and high sensitivity to some chemotherapy drugs. In conclusion, we constructed a signature composed of immune-related LncRNA pair with no requirement of the specific expression level of genes, which shows promising clinical predictive value in CRC patients.

## Introduction

According to global cancer statistics in 2020, colorectal cancer ranks third in cancer incidence and becomes the second leading cause of cancer deaths (Sung et al., 2021). And its incidence has been steadily increasing in many countries in Eastern Europe, Southeast Asia, Central-south Asia, and South America (Arnold et al., 2017, 2020). Although the diagnosis and treatment of colorectal cancer continue to make progress, the prognosis of advanced patients is poor, mainly due to recurrence, metastasis, and drug resistance (Nishihara et al., 2013). Moreover, the prognosis of patients in the same disease stage is different, accompanied by different gene mutations (Sinicrope et al., 2017). Genetic and molecular changes play an essential role in these events and provide potential targets for treatment (Sadanandam et al., 2013).

Immunotherapy accelerates the development of oncology and shows encouraging anti-tumor efficacy in many types of solid cancers (Farkona et al., 2016). Among various immunotherapy methods, immunomodulatory monoclonal antibodies (mabs) that target immune checkpoints have produced promising and long-lasting therapeutic results in multiple cancers. However, in colorectal cancer, the tumor has a high proportion of resistance and ineffectiveness to these monoclonal antibodies, such as anti-PD-1 mabs (Guo et al., 2020). Therefore, more alternative therapies and biomarkers used to predict the prognosis and treatment response of CRC need to be further investigated to allow more patients to benefit from immunotherapy.

Long noncoding RNAs, defined as RNA that transcriptional length is more than 200 nucleotides, do not encode proteins (Chan and Tay, 2018). LncRNAs interact with DNA, mRNAs, ncRNAs, and proteins to regulate gene expression at different levels, and play an essential role in both normal development and tumor progression (Ørom et al., 2010; Wang and Chang, 2011). LncRNAs are frequently involved in different stages of CRC from precancerous polyps to distant metastasis, and are considered potentially effective diagnostic biomarkers (Ye et al., 2015; Saus et al., 2016). Studies have shown that lncRNAs can also lead to cancer’s malignant progression by changing the tumor microenvironment (Atianand et al., 2017). LncRNAs regulate the gene-coded products involved in the immune response and affect the activation of immune cells, thus leading to the infiltration of immune cells in tumor (Chen et al., 2017).

Clinical predictive signatures focusing on immune-related markers have shown favorable diagnostic and predictive performance in various tumors. Shen et al. (2020) identified 11 lncRNAs associated with immune cell infiltration to construct the predictive signature of breast cancer. Wu et al. (2020) identified 8 immune-related LncRNAs and demonstrated their value in predicting the prognosis and immunotherapy response of bladder cancer. Hong et al. (2020) incorporated 12 pairs of immune-related LncRNAs into the model, which has good clinical predictive value in hepatocellular carcinoma.

As far as the accuracy of the cancer diagnosis signature is concerned, the combination of two markers is better than a simple single gene (Lv et al., 2020). For the simplicity and practicality of the signature, we tried to construct a reasonable model based on 2-lncRNA combinations (Hong et al., 2020; Chen et al., 2021; Ping et al., 2021). Compared with the single gene model that needs to detect the specific expression level of each marker, our model only needs to compare the expression level of each lncRNA pair and substitute 0 or 1 into the model. This will effectively avoid data correction during model application. We evaluated this signature’s predictive value in CRC patients, including survival rate, clinical progression, immune cell infiltration, and chemotherapy effects.

## Materials and Methods

### Retrieval and Preparation of Transcriptome and Clinical Data

The transcriptome data of colorectal cancer were downloaded from The Cancer Genome Atlas (TCGA, RRID:SCR_003193) database,^1^ and the data type was FPKM (fragments per kilobase million). The dataset includes 44 normal tissues and 568 tumor tissues. We downloaded the GTF (gene transfer format) file from Ensembl^2^ to distinguish mRNA and lncRNA. Clinical data of CRC patients were retrieved from the TCGA database. To extract valid data, duplicate data and data with a follow-up time fewer than 31 days were eliminated.

### Identification of Differentially Expressed Immune-Related LncRNAs (DEirlncRNAs)

The list of immune-related genes (ir-genes) was obtained from the Immport database,^3^ and the co-expression analysis was performed to screen immune-related LncRNAs. We analyzed the expression correlation between ir-genes and all lncRNAs. The screening criteria for irlncRNAs were immune gene correlation coefficient > 0.4 and *p-*value < 0.001. To identify DEirlncRNAs between normal and cancer tissues, R package limma was used to analyze the differential expression of irlncRNAs. We set thresholds as log2 |fold change| > 1 and false discovery rate (FDR) < 0.05.

### Pairing of DEirlncRNA

DEirLncRNAs were periodically paired to construct a 0-or-1 matrix. Suppose that C is equivalent to a pair of DEirncRNA, such as LncRNAA and LncRNAB. If the expression level of LncRNAA is lower than that of LncRNAB, then C is defined as 0; otherwise, C is defined as 1. Next, the matrix was screened further. If the expression of the DEirLncRNA pair is counted as 0 or 1 in most samples, this pair will not be used for subsequent prognostic analysis since gene pairs without a certain level of difference cannot accurately predict patients’ survival. When the number of DEirncRNA pairs of which expression quantity was 0 or 1 accounted for more than 20% and less than 80% of the total samples, the pair was considered to be an effective match.

### Construction of Risk Signature to Evaluate Risk Score

The DEirlncRNA pair was analyzed by univariate analysis, followed by lasso regression with 10 fold cross-validation, and the *p*-value was set to 0.05. Lasso regression ran 1,000 cycles to obtain the DEirlncRNA pair combination with the smallest cross-validation error, and then Cox proportional hazards regression analysis and model construction were carried out. We determined the optimal model according to the Akaike information criterion (AIC) value. When the AIC value was minimum, the calculation process was terminated, and the model was regarded as the optimal candidate. The receiver operating characteristic (ROC) curves of 1-, 3-, and 5-year were drawn afterward. The following formula can calculate the risk score of all cases: RiskScore = $\sum_{i=1}^{N}Expi*$Wi, where exp is the expression value of every DEirlncRNA pair, and W is the multivariate cox regression analysis coefficient of each DEirlncRNA pair in the signature. The sum of sensitivity and specificity of each point in the 5-year ROC curve was calculated, and the risk score corresponding to the maximum point was taken as the cut-off value to distinguish the risk level.

The R packages used in the above steps were survival, surviviner, survivalroc, and glmnet.

### Verification of the Constructed Risk Signature

We first used the LncAR database to verify the differential expression of the irlncRNA contained in the signature. To verify the cut-off value, Kaplan-Meier analysis was performed to show the survival difference between the high-risk and low-risk groups by using the survival curve. Using R tool, we also visualized the specific risk score of each sample in the signature.

For the sake of verifying the clinical application value of the signature, the chi-square test was used to analyze the relationship between the signature and clinicopathological characteristics. Afterward a band diagram was drawn for visualization (^∗∗∗^*P* < 0.001; ^∗∗^*P* < 0.01; ^∗^*P* < 0.05). Wilcoxon signed rank sum test was applied to calculate the difference of risk score among groups with different clinicopathological characteristics. The analysis results were shown by box diagram. Univariate and multivariate Cox regression analyses were performed to evaluate the correlation between risk score, clinical variables, and prognosis of patients, so as to clarify whether the risk model can be used as an independent prognostic indicator of colorectal cancer. *P* < 0.05 was considered statistically significant. The results were demonstrated by forest map.

The R packages used for the above analysis steps are survival, surviviner, survivalroc, limma, ggpubr and complex Heatmap.

### Analysis of Tumor-Infiltrating Immune Cells

To explore the relationship between the risk score and the tumor-Infiltrating immune cells, we used various currently recognized methods to evaluate the immune cell infiltration status of colorectal cancer, including XCELL, TIMER, QUANTISEQ, MCPCOUNTER, EPIC, CIBERSORT-ABS, and CIBERSORT. The Wilcoxon signed rank sum test was implemented to compare the content of infiltrating immune cells between the high-risk and low-risk groups. Through Spearman correlation analysis, the relationship between the risk score and infiltrating immune cells was analyzed. The threshold was *P* < 0.05, and the results were displayed in the lollipop graph. The R packages limma, scales, ggplot2, and ggtext were used for the analysis.

### Evaluation of the Model’s Role in Clinical Treatment

To evaluate whether the model has a certain application value in the clinical treatment of colorectal cancer, we calculated the half-inhibitory concentrations (IC50) of commonly used chemotherapy drugs in the TCGA data set. The anti-tumor medications used in the analysis included gemcitabine, Rapamycin, Imatinib, Lenalidomide, and Shikonin. Wilcoxon signed rank sum test was performed to compare the difference in IC50 of drugs between the high-risk and low-risk groups. The results were displayed in the form of the box plot.

The R packages used in this part include limma, ggpubr, pRRophetic and ggplot2.

## Results

### Recognition of Differentially Expressed Immune-Related LncRNAs (DEirlncRNAs)

**FIGURE 1:**
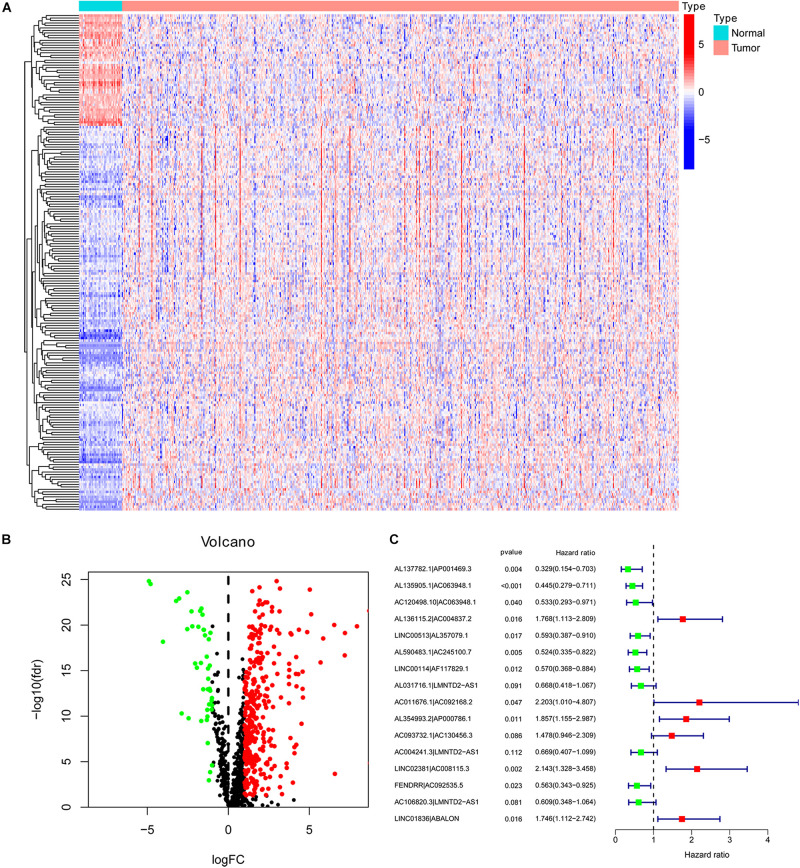
Identification of differentially expressed immune-related LncRNA (DEirlncRNA) and the construction of a signature. The expression information of DEirlncRNA was displayed in the heatmap **(A)** and volcano plot **(B)**. **(C)** A forest map showed 16 DEirlncRNA pairs included in the signature.

First, we downloaded the transcriptome data of colorectal cancer from the TCGA database. Then, the data were annotated based on the GTF file. Co-expression analysis between immune-related genes and lncRNA was performed. A total of 1017 immune-related lncRNAs were identified. Through differential expression analysis, 383 were classified as DEirlncRNAs, of which 339 were highly expressed and 44 were low expressed (Figure 1B). The expression of the DEirlncRNAs ranked in the top 200 based on fold change was displayed in the heat map (Figure 1A). The complete list of differentially expressed immune-related lncRNAs was shown in Supplementary Table 1.

### Construction of DEirlncRNA Pair and Risk Assessment Signature

Through the iterative loop and the construction and screening of a 0-or-1 matrix, 39528 valid DEirlncRNA pairs were obtained from 383 DEirlncRNAs. By univariate analysis, 4197 lncRNA pairs related to prognosis were identified. Subsequently, 26 DEirlncRNA pairs were extracted by LASSO regression analysis to prevent the model from over-fitting, of which 16 pairs were incorporated into the COX proportional hazard model by a stepwise method (Figure 1C).

**FIGURE 2:**
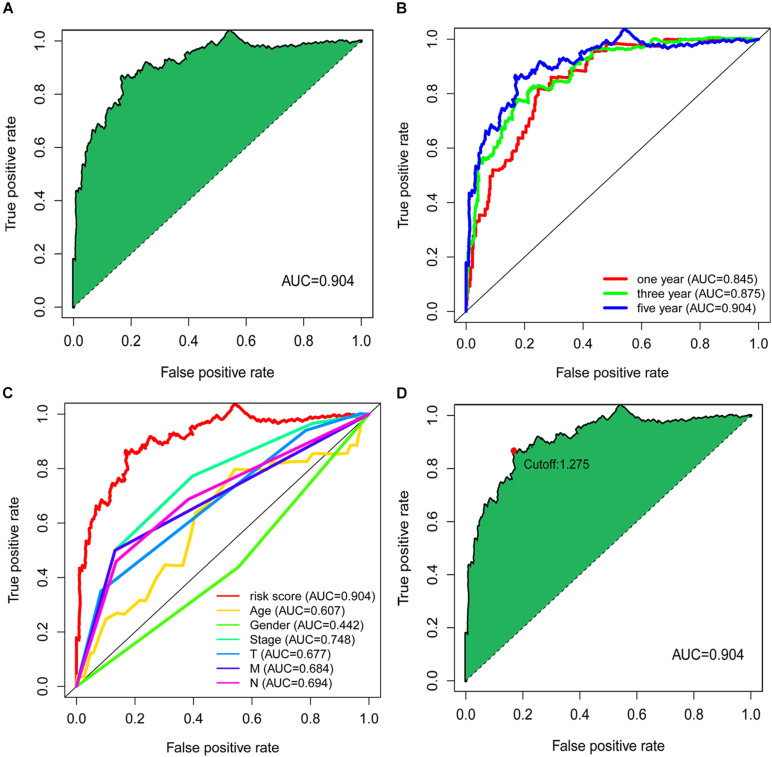
Establishment of the signature based on DEirlncRNA pairs. **(A)** The 5-year ROC of the optimal signature. **(B)** The AUC values of the 1-year, 3-year, and 5-year ROC curves of the model. **(C)** The comparation of 5-year ROC curve with other clinical characteristics. **(D)** The risk score of 1.275 at the maximum end was taken as the cut-off value to distinguish the high and low risk of samples.

The value of the area under curve (AUC) of the signature was 0.904 (Figure 2A), indicating an ideal predictive performance of the model. To verify the signature’s superiority, we plotted the 1-, 3-, and 5-year ROC curves, and the results showed that the AUC values of all three curves were over 0.80 (Figure 2B). We also compared 5-year ROC curve with other clinical characteristics, and the risk score had the most considerable AUC value (Figure 2C).

By calculating the sum of sensitivity and specificity of each point of the ROC curve for 5 years, the risk score of 1.275 at the maximum end was taken as the cut-off value to distinguish the high and low risk of samples (Figure 2D). The signature was applied to 500 colorectal cancer samples available from the TCGA database, and the risk score of these patients was calculated. Whereafter, these samples were divided into a high-risk group and a low-risk group by the cut-off point identified above for further validation.

### Validation of Risk Assessment Model and Its Application in Clinical Evaluation

**FIGURE 3:**
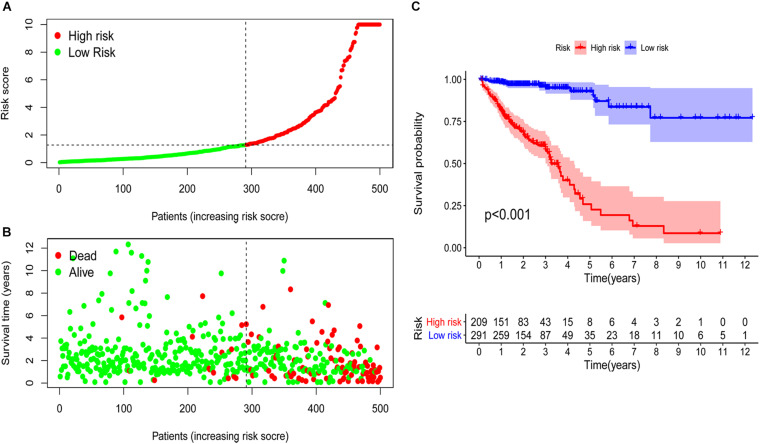
Risk assessment model for survival prediction. The risk scores **(A)** and survival time **(B)** of each case were shown. **(C)** The survival time of the high-risk group was significantly shorter than that of the low-risk group.

The external data validation of the expression of irlncRNA in the model was shown in Supplementary Figures 1, 2, and the detailed data sources were shown in Supplementary Table 2. Based on the cut-off value, 209 samples were classified into the high-risk group and 291 cases into the low-risk group. The risk scores and survival time of each case were shown in Figures 3A,B. The results showed that the survival rate and survival time decreased with the increase of the risk score. Survival analysis demonstrated that the survival time of the high-risk group was significantly shorter than that of the low-risk group (*p* < 0.001) (Figure 3C).

**FIGURE 4:**
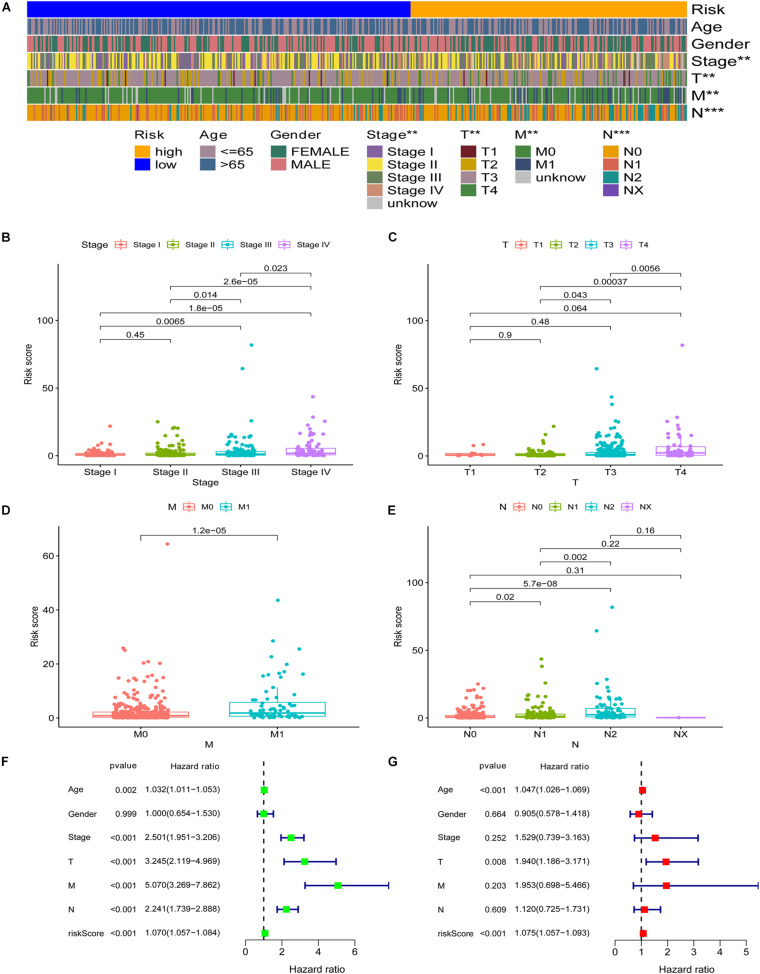
Application of the signature in the clinical evaluation. The band diagram **(A)** and scatter plots showed that clinical stage **(B)**, T stage **(C)**, M stage **(D)**, and N stage **(E)** were significantly associated with risk score. Univariate COX regression analysis showed that age, clinical stage, T stage, M stage, N stage, and RiskScore were considered statistically significant **(F)**. **(G)** Multivariate Cox regression analysis showed age, T stage, and RiskScore were independent prognostic predictors. ^∗∗∗^*P* < 0.001; ^∗∗^*P* < 0.01; ^∗^*P* < 0.05.

Subsequently, we applied the chi-square test to explore the relationship between the risk score and clinicopathological characteristics. The band diagram (Figure 4A) and scatter plots showed that clinical stage (Figure 4B), T stage (Figure 4C), M stage (Figure 4D), and N stage (Figure 4E) were significantly associated with risk score. In general, groups with advance stage were accompanied by higher risk scores. Univariate COX regression analysis showed that age (*p* = 0.002, HR = 1.032, 95% CI [1.011–1.053]), clinical stage (*p* < 0.001, HR = 2.501, 95% CI [1.951–3.206]), T stage (*p* < 0.001, HR = 3.245, 95% CI [2.119–4.969]), M stage (*p* < 0.001, HR = 5.070, 95% CI [3.269–7.862]), N stage (*p* < 0.001, HR = 2.241, 95% CI [1.739–2.888]), and RiskScore (p < 0.001, HR = 1.070, 95% CI [1.057–1.084]) were considered statistically significant (Figure 4F). Multivariate Cox regression analysis showed age (*p* < 0.001, HR = 1.047, 95% CI [1.026–1.069]), T stage (*p* = 0.008, HR = 1.940, 95% CI [1.186–3.171]), and RiskScore (*p* < 0.001, HR = 1.075, 95% CI [1.057–1.093]) were independent prognostic predictors (Figure 4G). The detailed information of univariate and multivariate Cox regression analysis was shown in Supplementary Table 3.

### Analysis of Immune Cell Infiltration Based on the Risk Score

**FIGURE 5:**
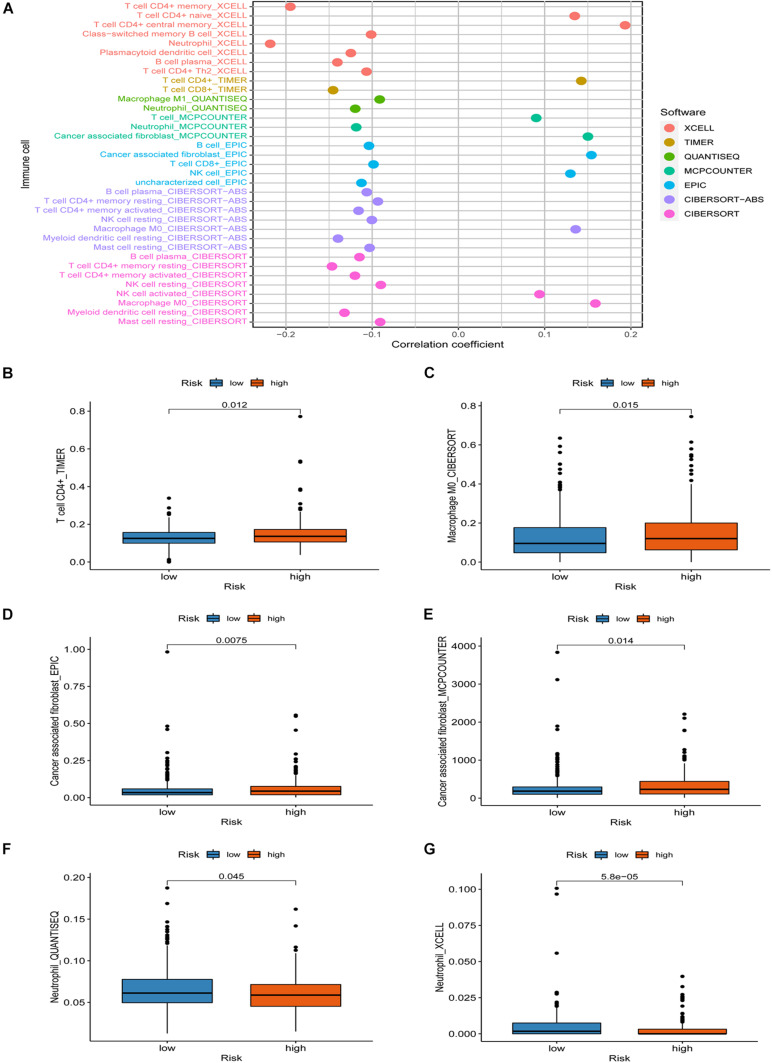
Association between immune cell infiltration and the risk score. **(A)** The relationship between risk score and immune infiltrating cells in multiple databases. **(B–G)** High risk was positively correlated with tumor-infiltrating immune cells such as CD4^+^ T cells, macrophage, and cancer-associated fibroblast, whereas negatively correlated with neutrophil.

Since the lncRNA identified by the co-expression method was related to immune genes, we explored whether the model was linked to the tumor microenvironment. The results showed that patients’ high risk was positively correlated with tumor-infiltrating immune cells such as CD4^+^ T cells, macrophage, and cancer-associated fibroblast, whereas negatively correlated with neutrophil (Figures 5B–G). Through Spearman correlation analysis, the relationship between risk score and immune infiltrating cells in multiple databases was displayed in Figure 5A.

### Correlation Between the Risk Model and Chemotherapy Drugs

**FIGURE 6:**
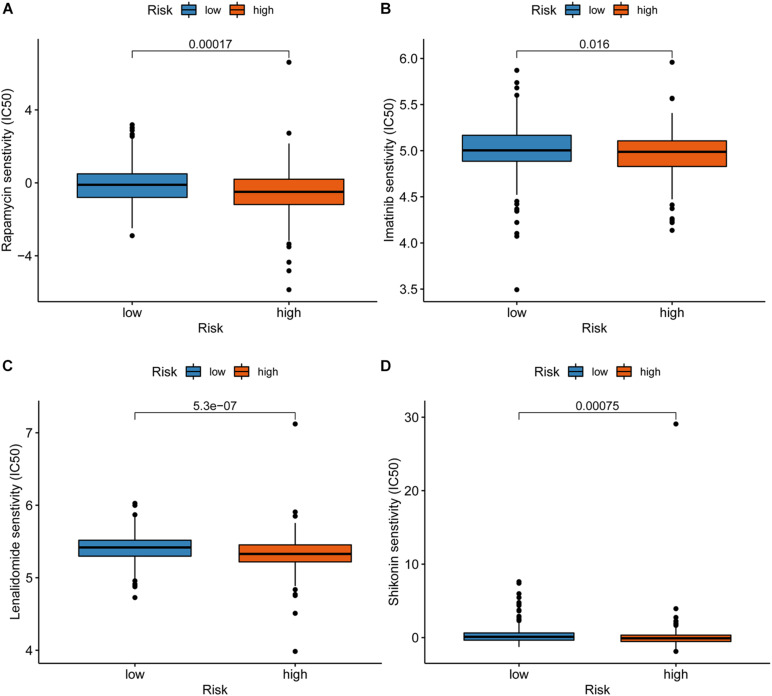
Correlation between the risk model and chemotherapeutics. A high-risk score was associated with lower IC50 for chemotherapeutics such as Rapamycin **(A)**, Imatinib **(B)**, Lenalidomide **(C)**, and Shikonin **(D)**.

We attempted to explore the relationship between the risk score and efficacy of common chemotherapy drugs for CRC in the TCGA dataset. The results showed that a high-risk score was related to lower IC50 of chemotherapeutics such as Rapamycin (*P* = 0.00017), Imatinib (*P* = 0.016), Lenalidomide (*P* = 5.3e-07), and Shikonin (*P* = 0.00075), suggesting that the model could be regarded as a potential predictor of chemotherapy sensitivity (Figure 6).

## Discussion

Recently, an increasing number of studies showed that infiltrating immune cells play an essential role in tumor management and become an effective prognostic factor for colorectal cancer (Picard et al., 2020). There were data disclosed that there seem to be subtle differences in the composition of immune cells infiltrated in colorectal cancer, which may be a key determinant of treatment and prognosis (Xiong et al., 2018). Besides, Pagès et al. (2018) have proposed that immune scores based on tumor-infiltrating immune cells can reliably estimate the risk of recurrence of colorectal cancer patients. These results support the view that the immune score could work as a new part of tumor TNM-immune classification. In recent years, many studies have focused on establishing immune-related coding genes and non-coding RNA signatures to assess the prognosis of colorectal cancer. However, most of the prognostic models are based on the quantified expression level of the sample. In this study, inspired by the gene pairing strategy, we tried for the first time in colorectal cancer to construct a reasonable model composed of paired lncRNA, which does not require the exact expression of lncRNA.

First, we obtained the original transcriptome data of colorectal cancer from the TCGA database, performed co-expression analysis and differential expression analysis to identify DEirlncRNA, and verified the effective DEirlncRNA pair by loop pairing and a matrix of 0 or 1. Second, we performed univariate analysis, LASSO regression analysis, and COX regression analysis to determine the DEirlncRNA pair for inclusion in the signature. Third, we determined the optimal signature by calculating the AIC value, and calculated the sum of sensitivity and specificity of each point on the 5-year ROC curve to find the best cut-off value. Finally, we evaluated the model from several aspects, including survival time, clinicopathological progress, distribution of tumor-infiltrating immune cells, and chemosensitivity.

At present, a number of studies have identified predictive biomarkers for colorectal cancer and have shown good clinical utility, which also provides ideas for the construction of more clinical models (Clarke et al., 2017; Martinez-Romero et al., 2018; Ahluwalia et al., 2019). Many studies suggested that lncRNA plays a non-negligible role in the development of colorectal cancer, and may participate in the remodeling of the tumor microenvironment and affect the infiltration of immune cells in the tumor. Qin et al. (2021) constructed an independent model based on 7 immune-related lncRNAs, which may promote the accurate assessment of the prognosis of CRC patients. Lin et al. (2020) identified a model containing 9 immune-related lncRNAs, which may help to improve the prediction results of colon cancer patients and guide individualized treatment. Li et al. (2020) constructed a seven immune-related lncRNA signature, which showed promising clinical significance in colon adenocarcinoma. Our algorithm showed that we could identify DEirlncRNAs and construct the most important irlncRNA pair for the first time in colorectal cancer. The model showed good predictive performance. The AUC values of the 1-, 3-, and 5-year ROC curves of the model were all above 0.80. What’s more, the most significant difference between our signature and the above-mentioned prognostic model is that the signature does not require each marker’s exact expression, but only needs to compare the expression level in each pair of DEirlncRNA. This dramatically improves the model’s clinical utility and largely avoids the error caused by differences in marker expression detection.

The DEirlncRNAs used to construct the signature in this study play critical roles in a variety of tumors. Dysregulation of FENDRR expression is associated with tumorigenesis, resistance to chemotherapy, fibrosis, and inflammatory diseases (Szafranski and Stankiewicz, 2021). In colorectal cancer, Yin et al. (2019) revealed that FENDRR could inhibit tumor aggressiveness by regulating the miR-18a-5p/ING4 axis. Data from Cheng et al. (2020) indicated that FENDRR could inhibit cell proliferation, migration, and invasion in CRC by targeting miR-424-5p. Liu and Du (2019) proved that FENDRR might work as a tumor suppressor gene in colon cancer by inhibiting SOX4. Also, FENDRR is closely related to immune regulation (Munteanu et al., 2020; Shen et al., 2021). Moreover, FENDRR takes effect in different cancers, such as hepatocellular carcinoma, cholangiocarcinoma, gastric cancer, cervical cancer, breast cancer, prostate cancer, endometrial cancer, and non-small cell lung cancer (He et al., 2018; Li et al., 2018; Qin et al., 2019; Yu et al., 2019; Zhang G. et al., 2019; Zhang Y.Q. et al., 2019; Zhu et al., 2020). It can be seen that FENDRR is closely involved in the process of tumors related to the digestive system and reproductive system. Lv et al. (2019) reported that LINC00114 promotes colorectal cancer by regulating the EZH2/DNMT1/miR-133bz axis, and Han et al. (2020) found that LINC00114 promotes the progression and radioresistance of nasopharyngeal carcinoma by targeting miR-203 to regulate the ERK/JNK signaling pathway. Jafarzadeh et al. (2020) indicated that LINC02381 might inhibit colorectal cancer tumorigenesis partly by regulating the PI3K signaling pathway. Besides, Jafarzadeh and Soltani (2020) also revealed that LINC02381 inhibits gastric cancer progression through the Wnt signaling pathway. In addition, LINC02381 plays a cancer-promoting role in cervical cancer and osteosarcoma (Chen et al., 2020; Bian et al., 2021). Some identified DEirlncRNAs were also included in the signatures of other colorectal cancer studies, such as LINC02381, LINC00114, and AL590483.1 (Wang et al., 2018; Li et al., 2020; Liu et al., 2020; Sun et al., 2020; Zhou et al., 2020), which verified the effectiveness of our algorithm. Another part of the selected DEirlncRNAs was reported for the first time. Therefore, the novel biomarkers need to be further explored.

We improved the modeling process, calculated the AIC value in Cox regression analysis to determine the best model, and compared the model with other clinicopathological characteristics. Instead of using the median value of risk score to distinguish the high and low risk of patients, we calculated the sum of sensitivity and specificity of each point on the ROC curve to find the optimal cut-off value. Then we re-evaluated the signature, and the results showed that its application effect was pretty good.

The occurrence and development of colorectal cancer involve many aspects of immunodeficiency. Tumor-infiltrating immune cells may affect the therapeutic effect of immune checkpoint inhibitors (Guo et al., 2020). To explore the relationship between risk score and tumor-infiltrating immune cells, we performed various methods to estimate infiltrating immune cells in colorectal cancer. Our signature was closely related to CD4^+^ T cells, CD8^+^ T cells, macrophages, cancer-associated fibroblast, and neutrophil through a comprehensive analysis. According to our signature, the high risk was associated with the sensitivity of chemotherapy drugs such as Rapamycin, Imatinib, Lenalidomide, and Shikonin. Given the limited drug data in the database, the sensitivity of more first-line chemotherapy drugs for colorectal cancer needs to be analyzed to further improve the signature’s practicality.

Our research also has shortcomings and limitations. First of all, we only obtained the original CRC data from the TCGA database, thus the number of samples may be relatively insufficient. We have not retrieved a useful dataset containing lncRNA expression levels and clinical information of CRC in other commonly used databases such as Gene Expression Ominibus (GEO). Since there were no data available, our model has not been externally verified. When a model based on the marker’s expression is validated with an external data set, due to the possible differences in sequencing on different platforms, the model’s effect may be affected. We constructed a 0-or-1 matrix to screen markers to minimize the errors caused by expression variations. Besides, we optimized the process of model construction and utilized various methods to verify the effectiveness of the signature. Based on the results, we believe that the signature we constructed is acceptable. However, the verification of the signature in a larger number of samples is still necessary. We will continue to collect samples in future clinical work and expand the verification scope for further evaluation.

## Data Availability Statement

Publicly available datasets were analyzed in this study. This data can be found here: https://tcga-data.nci.nih.gov/tcga/.

## Author Contributions

LX and MS designed the work. TZ and YW collected and integrated the data. MS analyzed the data and prepared the manuscript. TZ, YW, MS, and WH edited and revised the manuscript. All authors approved the final manuscript.

## Conflict of Interest

The authors declare that the research was conducted in the absence of any commercial or financial relationships that could be construed as a potential conflict of interest.

## Publisher’s Note

All claims expressed in this article are solely those of the authors and do not necessarily represent those of their affiliated organizations, or those of the publisher, the editors and the reviewers. Any product that may be evaluated in this article, or claim that may be made by its manufacturer, is not guaranteed or endorsed by the publisher.

## Footnotes

1
https://tcga-data.nci.nih.gov/tcga/

2
http://asia.ensembl.org

3
http://www.immport.org

## Abbreviations

CRC: Colorectal cancer
TCGA: The Cancer Genome Atlas
irlncRNA: Immune-related LncRNA
DEirlncRNA: Differentially expressed irlncRNA
AIC: Akaike information criterion
mabs: Monoclonal antibodies
FPKM: Fragments per kilobase million
GTF: Gene transfer format
ir-genes: Immune-related genes
FC: Fold change
FDR: False discovery rate
ROC: Receiver operating characteristic
IC50: Half-inhibitory concentrations
AUC: Area under curve
OS: overall survival
GEO: Gene Expression Ominibus.
