# Supplementary material for: A Novel Signature Constructed by Immune-Related LncRNA Predicts the Immune Landscape of Colorectal Cancer
Source: Front Genet. 2021 Aug 9;12:695130. doi: 10.3389/fgene.2021.695130 (PMC8381735; doi:10.3389/fgene.2021.695130)
Supplement: Supplementary file 1 [file Presentation_1.pdf]

Supplementary Material

Supplementary Figure 1. The expression of immune-related lncRNAs in the model in the LncAR database (the former 12 lncRNAs with expression information).

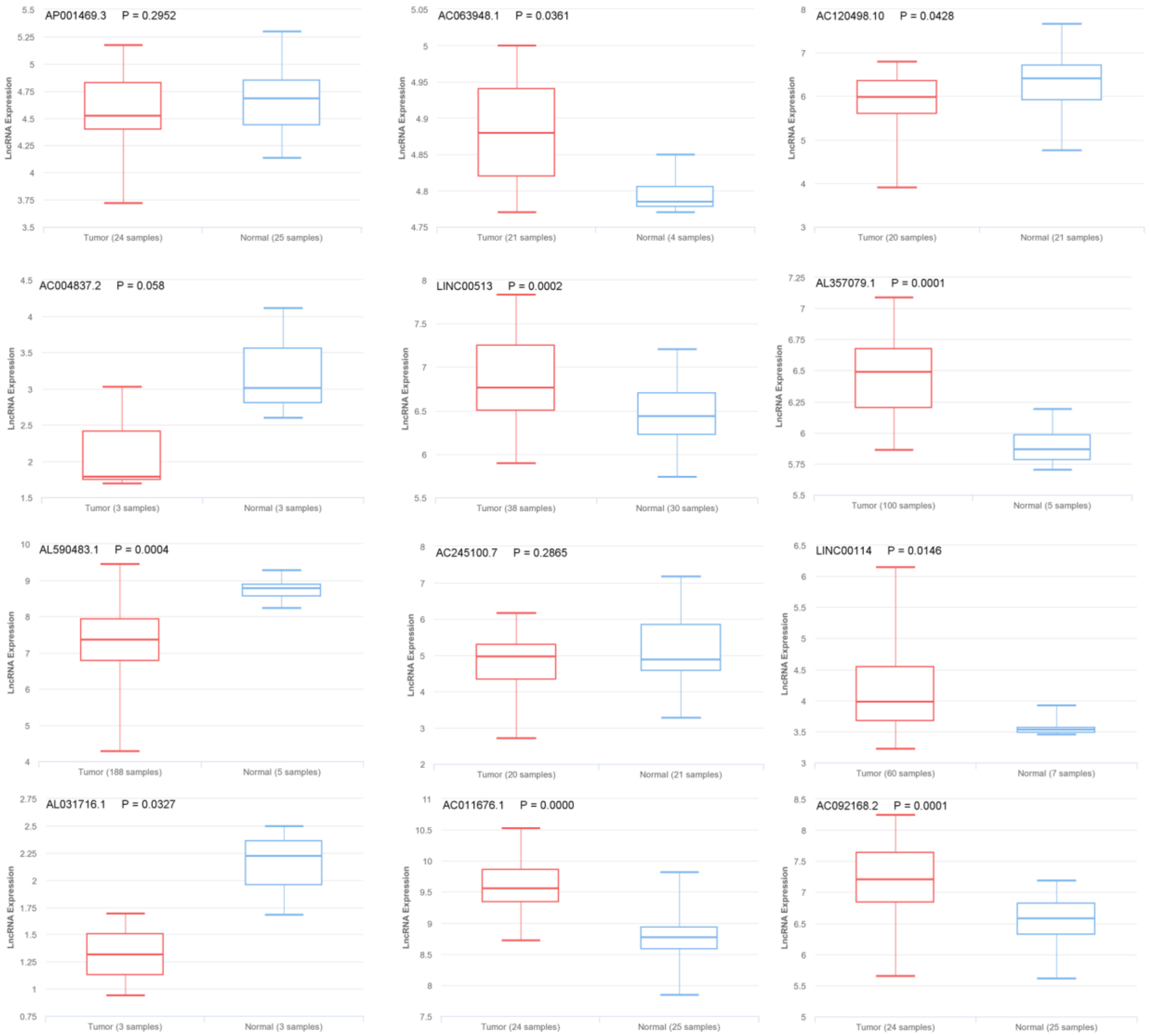

Supplementary Figure 2. The expression of immune-related lncRNAs in the model in the LncAR database (the last 11 lncRNAs with expression information).

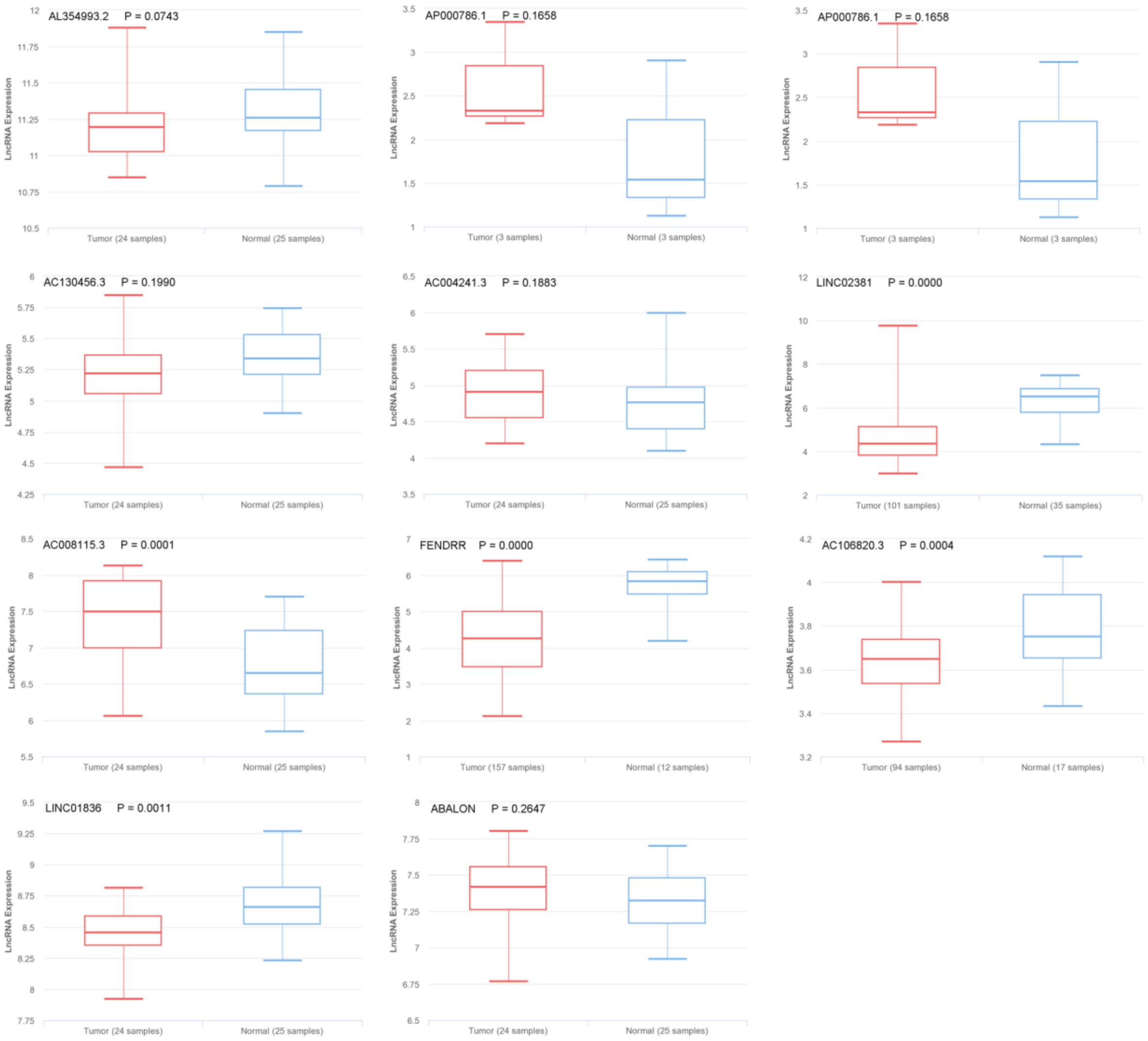

Supplementary Table 1. The complete list of differentially expressed immune-related lncRNAs.

Gene	ConMean	TreatMean	LogFC	P Value	FDR
AC015922.3	6.156797766	2.168664343	-1.505373405	1.76E-21	3.37E-20
LINC02441	14.87627703	3.07775514	-2.273063157	7.88E-24	3.14E-22
AL356299.3	0.103444682	0.83241028	3.008435302	1.73E-21	3.36E-20
AC007038.1	0.459911871	1.150128523	1.322365745	2.73E-08	7.04E-08
SCARNA9	0.467089376	6.259261053	3.74422181	7.99E-13	3.85E-12
ZKSCAN2-DT	0.412215337	1.029174468	1.320017482	2.64E-11	1.01E-10
AC112496.1	0.197595891	0.898771108	2.185400801	0.000142581	0.000230497
LINC01752	10.36491594	2.223832934	-2.220588102	5.04E-22	1.37E-20
AL137782.1	0.244623695	0.76248258	1.640140225	7.64E-14	4.35E-13
MIR3142HG	0.488773985	1.707880338	1.804967492	2.19E-09	6.55E-09
AC087277.2	1.740455247	0.725727277	-1.261965317	1.83E-14	1.13E-13
AC245884.8	0.356094987	1.193179113	1.744476598	2.69E-14	1.60E-13
AC124319.1	0.115125861	1.717182435	3.898759465	6.76E-21	1.09E-19
AL138689.1	0.298113041	1.098566547	1.881690874	2.80E-06	5.67E-06
TUSC8	0.844627148	3.279812648	1.957226883	1.85E-07	4.31E-07
ANKRD10-IT1	4.176647762	10.92686032	1.387461538	1.24E-06	2.65E-06
AC007342.5	0.53181223	1.315650736	1.306787689	2.35E-09	7.00E-09
AC145285.6	0.205136749	0.603137588	1.555901181	1.01E-13	5.63E-13
AP001160.1	0.200303302	0.762080203	1.927756637	7.05E-20	9.22E-19
AL021707.6	1.554888018	3.180559369	1.032469834	3.42E-08	8.67E-08
ARHGEF38-IT1	0.09825813	0.665388429	2.759548086	1.11E-09	3.46E-09
ACBD3-AS1	0.32604447	0.845378149	1.37452807	8.50E-06	1.57E-05
AC079907.1	0.240591003	0.57870333	1.266241253	4.31E-10	1.43E-09
AC010280.1	0.175607512	0.863263736	2.297446821	0.000417786	0.000635701
FTX	0.204885983	1.086627365	2.406964092	1.11E-05	2.01E-05
AC073487.1	0.208875847	0.599409314	1.520895821	3.87E-06	7.68E-06
MIR100HG	1.665738663	0.691670165	-1.268005942	3.61E-08	9.10E-08
LINC01355	0.387933809	0.998784925	1.364363531	3.67E-09	1.06E-08
AC025857.2	1.248648947	4.688752284	1.908836135	1.78E-20	2.51E-19
AL135905.1	2.361064709	0.820061365	-1.525633806	1.62E-21	3.28E-20
AC120498.10	1.206643959	0.541970653	-1.154713408	1.58E-14	1.00E-13
AC024060.2	1.752267933	3.628474501	1.050139742	2.15E-11	8.44E-11
AC004982.2	0.621060687	1.34671039	1.116633478	6.55E-14	3.75E-13
AC018653.3	0.440109002	1.147181949	1.382161444	2.11E-11	8.36E-11
AC016888.1	9.398811413	2.448393838	-1.940642681	5.50E-17	4.84E-16
AL022322.1	0.697897829	1.6505027	1.24181775	4.57E-09	1.31E-08
AL136115.2	0.054557879	0.665299353	3.60814416	2.75E-05	4.83E-05
AC091057.1	0.472543354	1.03143459	1.12613373	6.13E-13	3.04E-12
AC092723.1	3.929107232	0.421419573	-3.220872315	2.70E-25	2.25E-23
AL161729.4	0.165096975	0.794173981	2.266141407	9.87E-14	5.52E-13
MBNL1-AS1	2.974191102	0.513593108	-2.5337996	1.24E-21	2.78E-20

LINC00513	0.718008289	2.218054763	1.627222581	2.48E-08	6.45E-08
AC026356.1	0.18929247	0.792186615	2.065223307	5.61E-18	5.78E-17
AC000123.1	0.918793669	1.898297269	1.046893112	3.78E-07	8.53E-07
AC004837.2	0.126241337	0.707605757	2.4867614	9.62E-10	3.03E-09
MIRLET7BHG	0.198442428	0.546657269	1.461916095	1.25E-10	4.43E-10
LINC01614	0.018298787	0.948080443	5.695189523	1.32E-17	1.27E-16
AC062037.3	0.377747444	0.790097563	1.064608819	0.005543846	0.007306709
AL121832.2	1.154719909	3.487359005	1.594591939	3.66E-17	3.25E-16
DLEU2	0.341396002	1.20168888	1.815545361	3.48E-14	2.03E-13
AC019080.5	0.365778185	0.837273643	1.194730175	1.09E-08	2.96E-08
AL590483.1	0.042326296	0.571840398	3.755986381	1.98E-17	1.80E-16
AC004264.1	0.220446929	0.804339541	1.867373268	1.55E-17	1.46E-16
LINC00114	0.238630776	0.773445678	1.696519852	8.52E-07	1.86E-06
AL121832.3	0.218964973	0.831291738	1.924654767	9.79E-18	9.74E-17
AC243967.2	0.096377734	0.608098286	2.657532735	6.94E-08	1.69E-07
AC016831.1	0.094524665	0.844190542	3.158805932	7.74E-24	3.14E-22
SNHG11	2.005940736	5.388499264	1.425604545	1.17E-20	1.74E-19
MALAT1	8.033544081	114.8000051	3.836942307	9.09E-09	2.54E-08
AL031716.1	0.311399483	0.932568733	1.582443509	3.12E-11	1.18E-10
SNHG3	3.489689222	10.9742228	1.652948305	1.65E-21	3.28E-20
AC087222.1	0.122409929	0.570828785	2.221337502	2.79E-08	7.13E-08
AP001610.3	2.189187225	0.889468253	-1.299380324	4.84E-12	2.03E-11
AC114296.1	0.217999118	0.671328257	1.622696068	0.008477596	0.010891274
AC012073.1	0.605948665	1.224683723	1.015141737	2.38E-15	1.63E-14
AL031985.3	0.458451923	1.20456796	1.393673437	3.10E-19	3.82E-18
AL035461.3	0.894329038	2.297285529	1.361052555	3.21E-12	1.39E-11
MCF2L-AS1	2.4873948	7.73037466	1.635902827	2.31E-15	1.59E-14
AC099850.4	3.164584421	8.990132688	1.506326354	2.28E-20	3.12E-19
AC127024.5	0.679727248	2.090039941	1.620502651	3.01E-13	1.57E-12
AC011676.1	0.08983662	0.635186642	2.821805011	1.71E-17	1.58E-16
MIR17HG	0.154990287	1.37160194	3.145612137	5.04E-21	8.39E-20
TSPOAP1-AS1	0.336405223	0.997791862	1.5685388	9.59E-12	3.92E-11
B4GALT1-AS1	3.425197799	1.135990833	-1.592236111	2.00E-23	7.18E-22
AC092168.2	0.03859693	0.707965931	4.197121925	5.67E-08	1.40E-07
AL354993.2	0.186366021	0.626185225	1.748450623	0.001032175	0.001484258
AC022211.2	0.405621695	0.854347088	1.074687481	0.000154342	0.000247597
AP001469.3	0.365939286	1.082467266	1.564647184	7.11E-21	1.12E-19
AL035071.1	1.833236878	3.873441604	1.079222777	6.60E-10	2.14E-09
GK-AS1	0.243958523	0.986131656	2.015144381	0.000150954	0.000242803
AL390719.2	5.532327066	15.12592389	1.45106491	8.69E-12	3.58E-11
AC079684.2	0.278057004	0.957702238	1.784196495	1.91E-12	8.68E-12
AC063948.1	0.290186108	0.633441885	1.126233808	1.05E-06	2.27E-06
AL163953.1	0.075984605	1.08886779	3.840977832	4.53E-21	7.83E-20

AF117829.1	0.216371118	0.63111956	1.544405403	3.13E-13	1.62E-12
MIR222HG	0.680037062	1.567237048	1.204538126	2.19E-08	5.72E-08
AC020907.4	0.282818615	1.221427358	2.110619082	8.78E-16	6.34E-15
AL031186.1	0.411450848	0.877213881	1.092208546	2.64E-09	7.79E-09
AL118505.1	1.33378799	2.755880782	1.046984115	3.37E-06	6.74E-06
AC093732.1	0.030332259	0.561557543	4.210509015	8.23E-15	5.35E-14
AC091729.3	0.990068906	2.068571996	1.06303433	3.07E-12	1.35E-11
PRR7-AS1	0.096240233	0.623012608	2.694549317	7.81E-24	3.14E-22
AC123023.1	0.020892692	1.942590241	6.538839427	3.08E-22	9.74E-21
SNHG7	3.87669837	10.44972172	1.43056413	1.41E-19	1.79E-18
THUMPD3-AS1	1.231733176	2.575897583	1.064385467	3.75E-12	1.61E-11
LINC02163	0.002726869	1.261734647	8.853947507	1.95E-22	6.37E-21
AC015813.1	0.931582981	2.754400359	1.563982086	2.23E-11	8.71E-11
UCA1	0.781604156	15.61061218	4.319945165	3.12E-16	2.45E-15
LINC02487	0.304916208	1.197246067	1.973234952	8.57E-14	4.82E-13
LINC02747	13.86241306	5.870409991	-1.239645242	1.10E-15	7.86E-15
AC108134.3	0.395368106	2.421168359	2.614435002	1.07E-08	2.94E-08
AC093788.1	0.249830166	0.69336718	1.472671863	1.83E-08	4.87E-08
BCL2L1-AS1	0.088203356	0.878104804	3.315487693	0.029942529	0.036618634
AC008760.1	0.629623631	1.270922316	1.013314256	1.01E-09	3.14E-09
AC092119.2	0.449084872	1.069852368	1.2523517	7.26E-10	2.34E-09
AC090116.1	0.045283736	1.103411485	4.606834107	1.69E-13	9.03E-13
AL031600.1	0.265883821	0.886294367	1.736989953	1.80E-12	8.22E-12
AL117382.2	2.623771609	11.55203179	2.138432569	3.52E-11	1.33E-10
AC055717.2	0.002783818	1.538416858	9.110166127	4.70E-19	5.67E-18
AC073957.3	0.702461578	1.494432894	1.089106894	5.28E-07	1.17E-06
PABPC4-AS1	0.165811989	0.535226271	1.690600603	0.001039221	0.001492047
SNHG20	0.951239987	2.348160783	1.303649928	1.50E-20	2.17E-19
ZNF433-AS1	0.379073789	0.764107794	1.011297472	1.43E-14	9.13E-14
AC010719.1	0.655132376	3.495220025	2.415524924	1.00E-18	1.17E-17
AC002128.2	0.276671346	0.601949974	1.121470359	0.000660528	0.000977454
AL354836.1	0.933521548	2.915128896	1.642804447	1.12E-17	1.10E-16
AL591178.1	0.499924066	1.174674942	1.232480702	4.61E-05	7.96E-05
GAS5	23.76809646	60.05687843	1.337303125	3.13E-19	3.82E-18
NALT1	1.51908609	0.661608934	-1.199153012	0.000754729	0.001111466
AL355488.1	0.716559226	1.627624809	1.183610317	2.43E-07	5.63E-07
AL161891.1	0.140355098	0.570625697	2.023463253	1.01E-18	1.17E-17
CD44-AS1	0.16619946	1.091263441	2.715011826	6.16E-15	4.06E-14
AC135388.1	0	0.572354598	Inf	1.12E-12	5.19E-12
GABPB1-AS1	0.518319703	1.191472199	1.200831148	1.67E-07	3.94E-07
AL157838.1	0.255120154	0.576923435	1.177202995	6.91E-06	1.29E-05
AC015849.3	0.936528072	2.115427325	1.175554979	1.36E-06	2.89E-06
AC008610.1	0.788735242	1.803499667	1.193186145	6.04E-10	1.97E-09
AC006333.1	5.522027272	2.571642638	-1.102507837	3.95E-21	7.09E-20

AC005256.1	0.00613508	0.894298916	7.187531209	1.91E-18	2.14E-17
LINC00265	0.99205444	2.225509014	1.165644146	1.07E-12	5.07E-12
AC132872.3	0.870125745	1.898577926	1.125623405	1.09E-08	2.96E-08
USP30-AS1	5.136605144	2.101317603	-1.289520942	2.90E-11	1.11E-10
LENG8-AS1	0.993844323	2.40961162	1.277708843	2.56E-16	2.06E-15
AP001628.1	0.205144438	0.821491935	2.001606373	5.68E-10	1.86E-09
VPS9D1-AS1	1.263533931	8.226738012	2.702856093	1.85E-24	1.06E-22
AL136084.3	1.422517605	0.709965023	-1.002626653	1.40E-05	2.52E-05
SNHG22	0.170774218	0.758785888	2.151602665	9.53E-08	2.27E-07
SNHG17	2.752220431	13.3923524	2.282741468	4.22E-25	3.22E-23
LINC01232	0.331854441	1.171473729	1.819702117	2.89E-21	5.40E-20
AC015922.2	8.39699421	2.517667792	-1.737785062	5.75E-24	2.77E-22
AC005392.2	13.91950174	2.544289552	-2.451772799	4.62E-11	1.72E-10
LINC01389	3.01075791	1.473528257	-1.030851982	3.86E-13	1.98E-12
MED8-AS1	0.24661365	0.585969344	1.248572534	8.92E-10	2.82E-09
AC108727.1	0.096628017	0.536127154	2.472061744	0.00710711	0.009313467
LINC01811	0.005224687	0.768231406	7.200052434	4.23E-21	7.45E-20
PIK3IP1-AS1	0.211050992	0.531310344	1.331963187	1.97E-10	6.77E-10
AC104964.1	0.095792967	0.569814353	2.57250032	2.43E-06	5.00E-06
AP002336.2	0.161784287	0.543640257	1.748580799	4.15E-11	1.55E-10
SMIM25	0.661858049	1.755992007	1.407692543	2.95E-13	1.54E-12
AC004241.3	0.438783478	0.927796903	1.080299825	3.20E-08	8.16E-08
MIR22HG	12.05243274	3.821914505	-1.656956967	2.81E-24	1.52E-22
AC008735.2	1.335538661	4.630178618	1.79364611	2.45E-12	1.09E-11
AC084117.1	0.231257499	0.705419012	1.608980308	1.21E-05	2.18E-05
AC005550.2	1.419645631	0.7048275	-1.010188733	1.95E-13	1.02E-12
SNHG4	0.888647012	3.584851862	2.012231132	5.00E-21	8.39E-20
AL590723.1	0.205803336	0.614585333	1.578346969	0.001531008	0.002157543
AC092171.4	0.306193862	0.851896661	1.476233071	2.41E-10	8.19E-10
BX470102.1	0.948256948	5.649948956	2.574887891	1.53E-21	3.25E-20
AC010973.2	0.266507074	0.881051493	1.72505251	5.88E-18	5.99E-17
LINC01876	0.157499273	0.62023493	1.977469606	4.32E-05	7.49E-05
AC087741.1	0.313667124	0.819586774	1.385662378	3.82E-12	1.64E-11
SLC12A9-AS1	0.637834603	3.072030728	2.267938375	5.09E-22	1.37E-20
AC145423.2	0.114323803	0.609980347	2.415636941	1.63E-14	1.02E-13
WNT5A-AS1	0.347262527	0.714184707	1.040270503	0.003048765	0.004106866
PSPC1-AS2	0.384071168	0.930953003	1.277334673	1.31E-06	2.79E-06
AC011462.4	0.328542606	0.881015932	1.423087637	4.20E-12	1.79E-11
MNX1-AS1	1.062978761	4.365382129	2.037995176	3.27E-22	1.00E-20
AL133243.2	0.410318348	1.21423533	1.565232483	1.18E-05	2.14E-05
SNHG1	4.041191099	15.44003438	1.933823485	2.19E-25	2.00E-23
AC020765.2	0.421521272	0.907894317	1.106918931	8.56E-08	2.06E-07
CYTOR	0.949024315	2.657900353	1.485770061	1.52E-20	2.18E-19

AP002907.1	0.199221957	0.648531344	1.702799642	1.07E-08	2.94E-08
AL133410.1	0.35032037	0.916805833	1.387941344	1.83E-13	9.71E-13
AC018809.1	0.22014759	0.568679269	1.369144158	8.90E-07	1.94E-06
AC020558.2	0.321314879	0.678060756	1.07742676	9.91E-08	2.36E-07
AC084125.4	0.379459381	1.066511039	1.490881532	6.82E-09	1.93E-08
AP006621.4	0.26479227	0.613129941	1.21133185	6.86E-05	0.000115279
AC018695.4	0.220832054	0.544233828	1.301277037	3.62E-08	9.10E-08
AC010761.1	0.90511303	2.420136721	1.418918681	1.26E-17	1.23E-16
AC253536.6	0.415050862	0.874823559	1.07570393	1.90E-08	5.03E-08
AC018521.6	1.426268272	0.645790058	-1.143108234	3.13E-17	2.81E-16
LINC00174	0.526739298	1.231620532	1.225396821	7.12E-13	3.49E-12
ARHGAP27P1-BPTFP1-KPNA2P3	0.701009185	1.458625622	1.05710439	7.76E-08	1.89E-07
ASMTL-AS1	0.851265052	2.443083837	1.521023064	2.47E-11	9.57E-11
AL031275.1	0.084874323	0.61548261	2.858318028	1.68E-05	2.99E-05
MHENCR	2.244386201	8.817955971	1.974123325	1.30E-21	2.84E-20
LINC02381	1.995307025	0.735436862	-1.439937361	8.77E-21	1.34E-19
AC141002.1	0.257917998	0.546198708	1.08251345	0.000114895	0.000188609
UBXN10-AS1	5.050603351	0.686110439	-2.879943021	1.26E-11	5.05E-11
AC131971.1	0.053719732	0.651384868	3.599986202	8.51E-06	1.57E-05
LINC00941	0.023838826	0.572999976	4.587149957	1.57E-16	1.31E-15
HM13-IT1	0.519481398	1.601674714	1.624437184	7.90E-14	4.47E-13
NCBP2-AS1	0.227244053	0.56574946	1.315920762	5.02E-07	1.12E-06
AC005041.3	0.323995558	0.768550748	1.246166491	9.92E-10	3.10E-09
ZFAS1	17.89898797	65.17572696	1.864456751	9.50E-25	5.80E-23
LINC02604	0.795669	2.413861875	1.601102828	1.22E-15	8.69E-15
AC103706.1	0.157685751	0.817050716	2.373373337	1.63E-14	1.02E-13
MAFG-DT	0.376928732	3.434733364	3.187834432	5.33E-27	9.76E-25
PCAT14	0.008901571	0.850485457	6.578082757	0.000134337	0.000218955
AL034550.1	0.373630663	0.813148421	1.121905845	6.75E-05	0.000113686
AC131009.3	1.133160039	3.649810444	1.687469907	8.07E-21	1.25E-19
CCR5AS	0.99968695	2.236337651	1.161589735	5.02E-08	1.24E-07
AP000873.2	0.269305555	0.548352948	1.025860797	6.88E-07	1.51E-06
AL162595.1	0.35553746	0.842194764	1.244152333	2.90E-12	1.28E-11
AC124283.2	0.31865363	0.859989263	1.432329554	5.24E-13	2.64E-12
AC022150.4	0.205654005	0.63208608	1.619901879	0.022277175	0.027464188
PPP1R14B-AS1	0.741895809	3.230010337	2.122250287	2.04E-23	7.18E-22
AC007099.1	0.001945149	0.808738638	8.699649226	5.34E-24	2.72E-22
LINC01473	0.19888062	0.751749558	1.918349415	2.74E-10	9.28E-10
C2orf27A	0.300332378	1.093915588	1.864869496	3.81E-18	4.06E-17
LINC02595	0.046991245	0.96328998	4.357506266	1.56E-21	3.25E-20
UNC5B-AS1	0.178807089	0.756712561	2.081341455	0.00939067	0.012013762
AL592546.2	0.997820636	0.489513626	-1.027431486	4.47E-12	1.89E-11

LINC01235	0.232235403	0.614195727	1.403110554	1.68E-06	3.52E-06
AC007637.1	1.128238058	0.489919453	-1.203455025	2.66E-20	3.58E-19
AL021578.1	0.148348812	0.837551007	2.497183683	4.76E-13	2.41E-12
AC011468.1	0.629646611	1.264337393	1.005767253	1.43E-06	3.04E-06
AC011445.2	0.342429848	0.693823865	1.018761004	0.001613703	0.002260171
AC010542.6	1.035650453	2.576248858	1.314734805	1.03E-13	5.67E-13
AC020915.2	0.243719042	0.683511658	1.48774697	2.21E-05	3.91E-05
AC009065.3	7.630592597	16.93648602	1.150267568	7.82E-16	5.78E-15
AL355312.3	0.096635333	0.695693722	2.847829623	8.28E-08	1.99E-07
AC100814.2	0.765433799	1.535038693	1.003925509	1.99E-06	4.12E-06
AP000692.1	0.205229279	0.527410702	1.36169028	3.30E-06	6.62E-06
AL357079.1	0.476242698	0.985208531	1.048732149	2.48E-08	6.45E-08
AL162724.2	0.225269307	0.678466812	1.590627489	8.22E-05	0.000137146
CHN2-AS1	0.030884819	0.741424569	4.58533013	1.62E-14	1.02E-13
AC074117.1	0.950651838	2.063430063	1.118055563	1.80E-16	1.48E-15
AL391244.2	0.683030809	1.842430092	1.431587321	5.96E-21	9.75E-20
AC104695.4	0.271814857	1.741289671	2.679460005	3.61E-05	6.32E-05
AC048341.2	0.54527576	1.403539811	1.364012058	2.13E-11	8.42E-11
DLGAP1-AS2	0.698676068	3.696365034	2.403411607	4.59E-25	3.23E-23
AC016831.4	0.050554834	0.857861136	4.084823169	5.29E-07	1.17E-06
LINC01871	5.666956836	2.492760809	-1.184829757	1.09E-12	5.11E-12
Z83843.1	0.614862919	3.085592567	2.327210867	8.89E-06	1.64E-05
AL031673.1	0.403371773	1.24186487	1.622326165	4.23E-10	1.41E-09
AC004253.1	0.332953484	0.815379471	1.292150999	3.18E-07	7.25E-07
AL049539.1	0.04883684	0.656014801	3.7476866	4.55E-15	3.02E-14
AC138932.6	0.22137941	0.529850857	1.259065277	0.002040584	0.002815022
LMO7-AS1	0.082398926	0.723453775	3.134203397	1.32E-18	1.50E-17
B3GALT5-AS1	4.528784517	0.276414167	-4.034220429	5.10E-20	6.78E-19
AC004908.2	0.40805415	0.857714633	1.071737119	3.62E-07	8.20E-07
PTOV1-AS2	1.259250284	2.816155482	1.161161933	1.10E-12	5.12E-12
AC027796.4	0.400112858	1.047125949	1.387956086	2.47E-11	9.57E-11
SERTAD4-AS1	3.187663253	1.001425827	-1.670443662	9.46E-11	3.38E-10
LINC01082	7.435350081	1.288262151	-2.528974471	2.20E-26	2.51E-24
Z82243.1	0.188862932	0.868115332	2.20054715	4.16E-13	2.12E-12
AL133330.1	0.128313889	0.672296861	2.389421072	1.26E-13	6.87E-13
AC145207.8	0.262372299	0.528094242	1.009180003	0.001386736	0.001966331
NKILA	0.36055204	1.34387773	1.898122473	1.45E-12	6.65E-12
LINC00106	0.860454697	2.289738274	1.41201156	2.57E-09	7.60E-09
PLAC4	0.011314185	0.655361435	5.856086211	2.14E-20	2.97E-19
AL596223.2	0.113637672	0.551288145	2.2783654	1.08E-12	5.11E-12
AC125807.2	0.66681139	1.687883603	1.339864765	7.03E-18	7.07E-17
AC009509.2	1.886136439	0.584815425	-1.689380773	1.52E-17	1.45E-16
DIO3OS	1.437793491	3.211708254	1.159484369	0.005141462	0.006786137

AL161772.1	0.381108132	1.052171093	1.465097024	3.32E-14	1.95E-13
AC002116.2	0.418440258	1.049940508	1.327214018	6.78E-17	5.86E-16
Z94721.1	0.231637376	0.581149891	1.327042251	2.77E-08	7.10E-08
AC092718.4	2.789031477	7.927798659	1.507156106	8.90E-22	2.04E-20
AC092338.1	0.129593575	0.560970968	2.11393191	0.00801801	0.010344362
OGFRP1	0.398262319	0.84000658	1.076681642	6.53E-16	4.86E-15
AC021218.1	1.440334679	9.01519071	2.645953933	6.48E-22	1.53E-20
AP001554.1	3.981401965	0.486573199	-3.032547771	1.18E-25	1.20E-23
AL606834.1	0.382462973	0.781869992	1.031608652	5.62E-05	9.54E-05
LINC00578	1.279574888	0.588552705	-1.120421065	8.49E-05	0.000140664
AC016773.2	0.324746441	0.949822509	1.548344232	4.32E-15	2.89E-14
AL109614.1	0.289904689	1.321295238	2.188302292	1.99E-09	5.97E-09
GAPLINC	0.226368589	0.640833017	1.501274696	5.39E-12	2.26E-11
LINC01748	0.073178411	1.320868502	4.17392494	2.93E-16	2.31E-15
TFAP2A-AS1	0.042494627	0.567134607	3.738338847	6.25E-16	4.70E-15
AC087588.2	0.47801277	1.012273122	1.082477533	8.31E-05	0.00013834
PAN3-AS1	0.504601793	1.078362602	1.095625133	7.93E-09	2.23E-08
AL354696.1	0.578029706	1.288162145	1.156098658	8.31E-11	3.00E-10
LINC02362	1.432084864	0.489699504	-1.548148349	1.83E-14	1.13E-13
UBE2R2-AS1	0.175997958	0.597482056	1.763336696	1.28E-06	2.73E-06
AL117332.1	0.369717179	0.884924309	1.259131982	5.56E-13	2.78E-12
AL928654.2	1.125072898	2.55976313	1.185991833	1.67E-08	4.45E-08
AC124067.4	3.424995131	10.01950752	1.548637757	7.64E-13	3.72E-12
AC127024.4	0.393316701	1.359168514	1.788960983	1.70E-09	5.13E-09
AP002387.2	3.002503009	6.751452573	1.169032239	1.45E-09	4.46E-09
FENDRR	6.221173386	1.72823244	-1.847889448	6.31E-22	1.53E-20
AC007128.1	0.017422108	0.60416294	5.115946593	1.72E-23	6.57E-22
NEAT1	10.64975852	33.84086899	1.667945898	0.005875175	0.007721176
AC005261.1	2.934418221	6.203211679	1.079940854	7.57E-15	4.95E-14
RHPN1-AS1	0.194905576	0.851333959	2.126949812	3.01E-18	3.26E-17
AL139349.1	0.322780841	1.040977722	1.68931234	5.88E-10	1.92E-09
SCAT2	0.185941366	0.843438788	2.181435609	2.06E-15	1.43E-14
LINC01705	0.005050395	1.241469434	7.941436734	5.09E-22	1.37E-20
AP003352.1	0.835303614	2.435818988	1.544034342	1.09E-20	1.64E-19
AC090739.1	0.35317118	1.107600475	1.648998054	5.88E-05	9.95E-05
AC007773.1	0.166005131	0.670573706	2.014168075	6.58E-17	5.74E-16
TRIM31-AS1	1.353005812	2.934184753	1.116791678	5.39E-10	1.78E-09
SNHG32	22.70862183	55.833252	1.297884435	6.53E-22	1.53E-20
AL031670.1	0.355122639	0.783851805	1.142263589	0.001145467	0.001639449
AL158837.1	1.152144318	0.483710876	-1.252104558	6.91E-17	5.92E-16
AC020656.2	0.033568999	0.613229631	4.191225978	3.02E-18	3.26E-17
AC004585.1	0.288319085	0.76756364	1.412620035	1.20E-13	6.57E-13
PGM5-AS1	5.259847724	0.176191167	-4.89980753	3.01E-28	1.49E-25
ZNF710-AS1	3.719725325	1.858667347	-1.000927504	1.38E-12	6.35E-12

AL442125.2	0.183503841	0.525545536	1.518005508	0.000575612	0.000860131
AC010536.2	0.2426708	0.826488965	1.767995032	0.002758779	0.003754891
AP005899.1	0.231374018	0.611003036	1.400952682	0.000404238	0.000617137
AC105460.1	0.009995083	4.284598969	8.743725927	7.83E-06	1.46E-05
AL133520.1	0.763901331	1.649581842	1.110642146	2.50E-09	7.45E-09
AC007938.3	0.269792073	0.773165786	1.518929837	9.71E-12	3.93E-11
SNHG15	2.231144557	8.47333797	1.925146424	3.17E-27	7.26E-25
AL445222.2	0.187793118	0.678019531	1.852182641	1.80E-13	9.60E-13
AC106820.3	0.215980909	0.588006585	1.444928515	4.60E-10	1.52E-09
AL355075.2	0.396882668	0.853300709	1.104341686	1.07E-05	1.95E-05
AGAP2-AS1	2.274878718	5.186466046	1.188962218	9.10E-13	4.34E-12
AC004816.1	1.047642971	2.169362311	1.050123881	9.53E-12	3.91E-11
AC121761.1	0.272132216	0.991513321	1.865324399	9.14E-15	5.90E-14
AC000061.1	0.163748582	0.863968186	2.399495773	6.37E-06	1.20E-05
LINC01836	0.108011197	0.761578718	2.817812287	1.90E-19	2.38E-18
ZDHHC20-IT1	0.11550813	0.58424947	2.33859012	2.64E-12	1.17E-11
N4BP2L2-IT2	0.254623894	0.847916756	1.735554827	4.15E-06	8.17E-06
AC078860.2	0.075679298	0.719692977	3.249410962	1.64E-13	8.89E-13
AC245100.7	0.204149486	0.606518571	1.570925883	2.52E-06	5.13E-06
LINC01311	0.244642708	0.881516193	1.849310792	7.77E-20	1.00E-18
CDKN2B-AS1	10.64837158	0.382549493	-4.798842602	1.02E-27	3.11E-25
AC022144.1	0.143005833	0.72014736	2.332218153	6.18E-16	4.68E-15
AL031714.1	0.492920608	0.990407793	1.006667368	3.06E-10	1.03E-09
AL513327.1	0.272949763	0.586625324	1.103803909	0.000130476	0.000213041
TMEM147-AS1	0.621753509	2.349008669	1.917637387	4.18E-23	1.42E-21
SNHG25	0.956366398	27.93002097	4.868109404	3.22E-21	5.90E-20
MCM3AP-AS1	0.324674574	0.6546964	1.011831637	1.99E-14	1.21E-13
AC058791.1	0.194254054	1.152535106	2.568794081	0.000304565	0.000471252
AC130456.3	0.444851027	0.978679237	1.137513811	3.96E-06	7.83E-06
SNHG16	2.115072192	5.892497307	1.478172288	1.45E-26	1.90E-24
LINC-PINT	0.517062841	1.580720093	1.612170391	1.13E-12	5.24E-12
AC136475.3	0.576941902	7.833329014	3.763127602	1.02E-16	8.60E-16
AC137630.3	0.237969575	0.659052556	1.469616386	0.001028394	0.001481147
AC008115.3	0.740413527	1.908128466	1.365755147	8.41E-11	3.02E-10
PVT1	0.382571416	3.025993383	2.983607841	3.25E-28	1.49E-25
AC093585.1	0.174027597	0.552469616	1.666579016	0.003606061	0.00482917
DTNB-AS1	2.013080522	0.486556695	-2.048725053	1.80E-17	1.65E-16
AC016394.3	0.467650199	1.616458168	1.789334466	7.59E-19	9.03E-18
AP006621.2	0.635972958	1.517523961	1.254681969	2.85E-06	5.77E-06
AC090152.1	2.234640436	0.88828118	-1.330954384	2.68E-18	2.96E-17
GIHCG	1.339867255	0.618077901	-1.116229487	2.69E-14	1.60E-13
MIR4435-2HG	0.619524408	1.944757249	1.650357057	7.74E-24	3.14E-22
CASC19	0.129479565	4.233542572	5.031069064	8.36E-27	1.28E-24

AL442067.1	0.127271595	0.733217073	2.526329912	2.24E-08	5.86E-08
LINC01138	0.410355363	0.87133614	1.086355574	2.58E-14	1.55E-13
MIR181A2HG	0.140556958	0.627433245	2.158307104	1.05E-08	2.91E-08
ABALON	0.183813014	0.790088932	2.10377614	7.90E-25	5.17E-23
AL590617.2	0.569798538	1.3038038	1.19420296	8.29E-16	6.03E-15
AP001429.1	0.055914166	0.946643548	4.081535537	1.06E-07	2.52E-07
AC108058.1	0.27363099	0.693951668	1.342603552	0.027636477	0.033888907
ALG13-AS1	0.345308436	0.990283017	1.519955317	0.040464032	0.048834062
AC109322.1	0.623490008	1.34360722	1.107673108	3.49E-15	2.35E-14
AC020916.1	6.175078459	15.58201071	1.335352039	2.62E-12	1.17E-11
AC008649.2	0.060111202	0.952784855	3.98644471	0.001418627	0.002008443
AL139089.1	0.380149716	1.47811783	1.959121661	1.60E-17	1.49E-16
AC132192.2	0.408665515	1.040325756	1.348042936	6.04E-14	3.48E-13
AC053503.3	3.675246955	1.467379359	-1.324599297	7.32E-11	2.65E-10
LINC01558	0.583417401	1.643221809	1.493926912	1.45E-06	3.07E-06
LINC02418	0.008056092	4.386432791	9.088752269	5.96E-22	1.52E-20
AC080129.2	0.411304121	1.191273453	1.534227184	1.60E-09	4.87E-09
Z68871.1	0.232272563	0.616639002	1.408607396	1.07E-08	2.94E-08
AC010834.3	0.459784651	1.139619751	1.309522319	3.36E-07	7.63E-07
AL078587.1	0.104905854	0.711036197	2.760827825	2.20E-12	9.90E-12
AC098851.1	0.272841134	0.58892618	1.110025644	0.040635213	0.048976125
AC092535.5	0.752063686	2.239269592	1.574101487	3.36E-13	1.73E-12
AP001042.1	0.123588888	0.55248425	2.1603843	3.77E-09	1.08E-08
AL512274.1	0.583305552	1.361689885	1.223074466	4.41E-07	9.87E-07
AL138963.1	0.091622475	0.86094834	3.232153231	0.000249768	0.000391759
AC008750.4	0.110903697	0.557317039	2.329190797	0.038950418	0.047131549
AC026368.1	0.075809933	0.617709711	3.026470222	2.89E-16	2.30E-15
AP000786.1	0.203193965	0.617265177	1.603032851	0.00991487	0.012666696
AP005271.1	0.028769046	0.659261794	4.518262174	2.00E-10	6.87E-10
AL117379.1	0.467001065	1.615812047	1.790761647	3.09E-12	1.35E-11
AC008870.2	0.275498929	0.668672754	1.279253625	6.30E-11	2.29E-10
AC006042.1	1.58637918	3.636590826	1.196848961	3.72E-08	9.34E-08
LINC01315	1.097878412	4.277659234	1.962103272	4.15E-18	4.37E-17
AL157786.1	0.466580329	0.945355791	1.018731916	0.026053217	0.032033217
LMNTD2-AS1	0.24776018	0.798837372	1.688957492	5.31E-11	1.94E-10
AC073611.1	0.41244519	1.150685382	1.48021911	5.55E-22	1.45E-20
FMR1-IT1	0.344688842	0.758895008	1.138605709	0.001560056	0.002195101
RUSC1-AS1	0.792767929	2.301291931	1.537473503	1.53E-15	1.07E-14
HIF1A-AS3	0.180900434	1.646950155	3.186529117	0.00477911	0.006326105
AC103591.3	0.353947319	1.561227912	2.141074607	2.67E-07	6.15E-07

Supplementary Table 2. Data source for irlncRNA expression verification in LncAR database.

Gene	Analysis ID
AL137782.1	None
AP001469.3	CR_S198
AL135905.1	None
AC063948.1	CR_S111
AC120498.10	CR_S217
AL136115.2	None
AC004837.2	CR_S56
LINC00513	CR_S161
AL357079.1	CR_S99
AL590483.1	CR_S6
AC245100.7	CR_S217
LINC00114	CR_S177
AF117829.1	None
AL031716.1	CR_S56
LMNTD2-AS1	None
AC011676.1	CR_S198
AC092168.2	CR_S198
AL354993.2	CR_S198
AP000786.1	CR_S56
AC093732.1	CR_S278
AC130456.3	CR_S198
AC004241.3	CR_S198

LINC02381	CR_S14
AC008115.3	CR_S198
FENDRR	CR_S36
AC092535.5	None
AC106820.3	CR_S128
LINC01836	CR_S198
ABALON	CR_S198

Supplementary Table 3. The detailed information of univariate and multivariate Cox regression analysis.

Covariates	Univariate analysis		Multivariate analysis	
	HR (95%CI)	*P*	HR (95%CI)	*P*
Age	1.032 (1.011-1.053)	0.002	1.047 (1.026-1.069)	0.000
Gender	1.000 (0.654-1.530)	0.999	0.905 (0.578-1.418)	0.664
Clinical stage	2.501 (1.951-3.206)	0.000	1.529 (0.739-3.163)	0.252
T stage	3.245 (2.119-4.969)	0.000	1.940 (1.186-3.171)	0.008
M stage	5.070 (3.269-7.862)	0.000	1.953 (0.698-5.466)	0.203
N stage	2.241 (1.739-2.888)	0.000	1.120 (0.725-1.731)	0.609
RiskScore	1.070 (1.057-1.084)	0.000	1.075 (1.057-1.093)	0.000
